# An Analysis on Promoting Prefabrication Implementation in Construction Industry towards Sustainability

**DOI:** 10.3390/ijerph182111493

**Published:** 2021-10-31

**Authors:** Zezhou Wu, Lirong Luo, Heng Li, Ying Wang, Guoqiang Bi, Maxwell Fordjour Antwi-Afari

**Affiliations:** 1 Sino-Australia Joint Research Centre in BIM and Smart Construction, College of Civil and Transportation Engineering, Shenzhen University, Shenzhen 518052, China; wuzezhou88@gmail.com (Z.W.); luolirong2018@email.szu.edu.cn (L.L.); 2 Department of Building and Real Estate, The Hong Kong Polytechnic University, Hong Kong, China; heng.li@polyu.edu.hk; 3 Jinan Haiying Real Estate Development Company, Jinan 250000, China; guoqiang5663@163.com; 4 Department of Civil Engineering, College of Engineering and Physical Sciences, Aston University, Birmingham B4 7ET, UK; m.antwiafari@aston.ac.uk

**Keywords:** prefabrication, sustainability, promotion policy, construction management, NVivo

## Abstract

As a game-changing technology with significant environmental, economic, and social benefits, prefabricated technology has attracted attention and has been increasingly adopted in the construction industry. Although multitudinous studies have investigated various aspects of prefabrication in construction, a thorough review of its current development state that synthesized environmental, economic, and social sustainability dimensions remains overdue. Therefore, this study aims to fill this research gap by constructing a systematic framework, analyzing the research status quos, and providing recommendations for future research. This study first conducted a holistic review of 768 references with NVivo. A research foci framework that represented the body of knowledge in prefabrication in construction was developed with five levels, which were advantages, hindrances, stakeholders, promotion policies, and strategy spectrum. Following the framework, the in-depth analyses from the perspectives of environmental, economic, social sustainability, technologies development, and promotion strategies were performed. The current research domains were further linked with potential research directions for promoting prefabricated construction towards sustainability. The study is of value in both offering references for policy formulation and stakeholder practice and providing recommendations for future research.

## 1. Introduction

The construction industry contributes significantly to global economic growth. However, its rapid development also produces adverse effects on the environment. According to the International Energy Agency, the most energy consumption and CO_2_ emissions come from the building industry [1]. Besides severe environmental damage, conventional construction methods could also cause economic and social issues, such as long construction periods, low labor productivity, and a high frequency of safety accidents [2]. With requirements of the low-carbon development model of modern society put forward, conventional onsite construction is no longer suitable for sustainable construction [3]. Thus, prefabrication has been introduced in the construction industry.

Prefabrication refers to a process of transporting off-site manufactured components to the construction site and then installing them to the buildings [4]. With the application of prefabrication, the construction waste can be reduced by 50% [5], resource reduction by 35.82%, health damage reduction by 6.61%, and ecosystem damage reduction by 3.47% [6]. Therefore, prefabrication application has been widely identified as a prospective way that contributes to the sustainable development of the construction industry [7].

Under the background of the sustainable development of the global construction industry, numerous researchers have explored the implementation of prefabrication in construction. The hot research topics include the identification of the factors that are driving or influencing prefabrication development [8,9,10,11,12,13]; the performance of prefabrication application, such as environmental sustainability [14,15], high capital cost [16,17], schedule risk [18,19], safety concerns [20,21]; and policy for promoting prefabrication development [22,23,24]. Buildings and their relevant construction processes can be evaluated by three critical dimensions of sustainability, i.e., environmental, economic, and social [25]. However, most researches on the current state of prefabrication implementation have mainly concentrated on one dimension of sustainable development [26,27,28] and lacked a comprehensive analysis that includes different sustainability dimensions. Therefore, this study aims to fill this research gap by constructing a systematic framework and providing recommendations for future research.

The following section introduces a selection of research methods. A framework is developed to understand the implementation of prefabrication in Section 3. Then, an in-depth discussion of existing studies from the perspectives of environmental sustainability, economic sustainability, social sustainability, promoting strategies, and future research directions is performed in Section 4. Lastly, the conclusion is presented in Section 5.

## 2. Materials and Methods

Currently, popular databases for retrieving papers are Scopus, Web of Science (WoS), and Google Scholar. Falagas et al. [29] stated that WoS has the highest coverage in the engineering field. Liu et al. [30] and conducted bibliographic analyses and proved that WoS was the priority choice for review studies in the prefabricated construction field. Hence, WoS was adopted in this study to collect papers. The retrieve timespan of this research was selected from 1 January 1990 to 31 December 2020 for two reasons. First, prefabricated building has become a hot topic since the 1990s. Second, the study aims to explore the current research status and discuss future directions; thus, studies before the 1990s were too old for achieving the objective. The topic search was used during the paper retrieving process, with the retrieval model: (TS = (“off-site construction” OR “off-site manufacturing” OR “prefabricated construction” OR “prefabricated building” OR “modular building” OR “precast building” OR “industrialized building”)) AND LANGUAGE: (English); Indexes = SCI-EXPANDED, SSCI, A&HCI, CPCI-S, CPCI-SSH, ESCI, CCR-EXPANDED, IC Timespan = 1990–2020. Initially, a total of 16,883 publications were captured.

After the collection of potentially related publications, two rounds of screening were then implemented. The first step is to filter out irrelevant data types and reserve only the article. The second step is to identify how the collected papers match the research scope by scanning titles, keywords, and abstracts. As the scope of this study is reviewing prefabrication in the construction industry, papers on prefabrication in other fields have been excluded. Finally, a total of 768 articles were collected for further analysis.

## 3. Results

The framework of prefabricated implementation in the construction industry was developed based on the content analysis of the captured articles with the assistance of NVivo.

### 3.1. Analyzing Contents Using NVivo

Given a large number of articles, it is appropriate to select computerized tools to analyze instead of manual analysis. According to existing studies, NVivo 11, which can conduct an exemplary content analysis of PDF format files, is a powerful software for qualitative research [31]. Notably, its functions of “Code” and “Model” enable users to deal with thousands of pieces of information, as well as clarify their relationships. Therefore, NVivo software was adopted in this study.

“Sources” are identified as all articles imported into NVivo, which were analyzed with the help of the “Node” function. References related to the same theme were categorized into the corresponding node called “coding” [32,33]. Using the sentence “the higher initial investment impeded the adoption of prefabrication” as an example, a two-level node structure was generated after screening this sentence. The second-level node is “Higher initial investment”, which was incorporated into the first-level node “Hindrance”. Then related references were coded under the corresponding node. When editing the nodes, the research boundary was severed as a useful reference, and human brains were used to determine the affiliations of all nodes in terms of specific themes [32]. To ensure the reliability and validity of the data, several rounds of coding were conducted manually [33].

**Figure 1:**
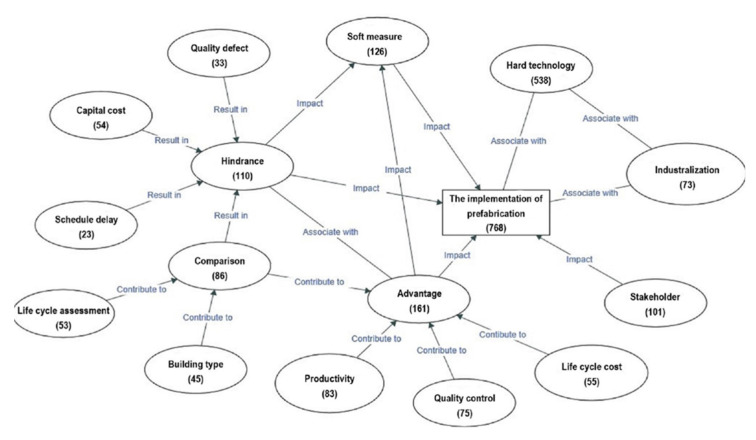
A tentative framework.

Next, “Model” could be used to develop a tentative framework based on the relationship between the nodes. In the tentative framework (see Figure 1), the rectangle represents the boundary of this research, which is “The implementation of prefabrication”, while the ellipse means the node generated in the coding process. The various arrows between two shapes indicate different relations, such as “Associate with”, “Impact”, “Result in”, and “Contribute to”. In addition, the number in each shape represents the total number of papers related to the specific theme and suggests the specific relationship between the two themes. It is worth noting that a paper might have more than one theme and relationship.

### 3.2. Developing a Framework of Prefabrication Implementation Research

**Figure 2:**
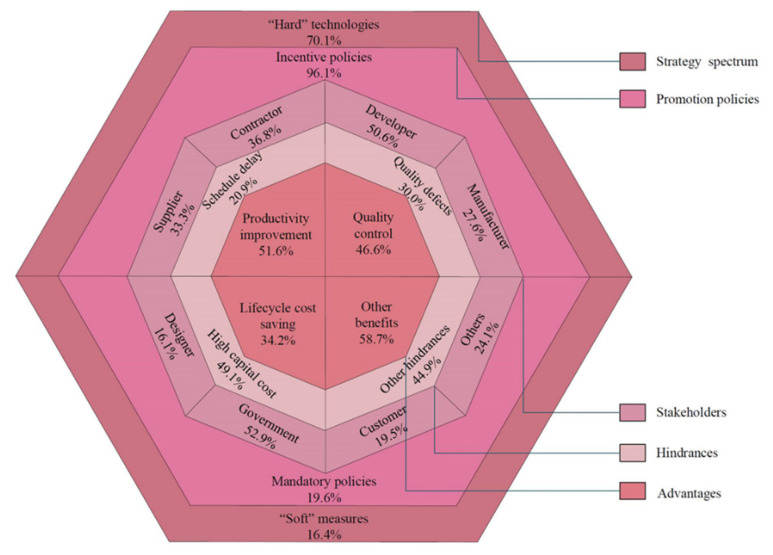
Prefabrication implementation research framework.

To better analyze the existing studies, a systematic framework of prefabrication implementation research with a five-level structure was developed, as shown in Figure 2.

This framework concludes five major components: (a) the “Advantages” presenting the benefits for adopting prefabrication; (b) the “Hindrances” indicating the obstacles of the adoption of prefabrication; (c) the “Stakeholders” revealing stakeholders’ attitudes and behaviors toward prefabrication; (d) the “Promotion policies” stating the policies being formulated by the government for promoting prefabrication; (e) the “Strategy spectrum” referring to the approaches including the “Hard technologies” and “Soft measures”. In those components, components (a), (b), and (c) were identified according to the second-level nodes in Figure 2. Component (d) was obtained from reviewing all nodes proposing policies; component (e) was summarized by the above components. Furthermore, the existing research can be examined from more than one perspective. This systematic framework helps researchers to grasp a general picture of existing studies of prefabrication implementation.

Similar to components (a), (b), and (c), many articles cover more than one theme, leading to the summation value of all factors in component (d) overrunning 100%. However, it is demonstrated that the summation value of all factors in component (e) is less than 100%. The research on prefabrication implementation can be understood by putting them into a “Strategy Spectrum” ranging from “hard” technology to “soft” measures. On one side of this spectrum are the “hard” technologies, referring to construction technology or structural performance. On the other side of this spectrum are the “soft” economic and managerial measures. Other researches, such as design systems and algorithm optimization, were excluded from both technical and managerial aspects of prefabrication implementation. In addition, the percentage of “Incentive policies” is higher than “Mandatory policies” in component (d), and the percentage of “Hard technologies” is higher than “Soft measures” in component (e). These results suggest that the investigation on “Mandatory policies” and “Soft measures” should be paid more attention in future research.

## 4. Discussion

The research foci shown in Figure 2 were further incorporated into three dimensions, which were environmental sustainability, economic sustainability, and social sustainability, with in-depth discussions. The construction technologies development and strategies for promotion were also discussed.

### 4.1. Environmental Sustainability

Previous research has demonstrated the environmental sustainability performance of prefabrication applications, including construction waste reduction, energy and resources saving, and air pollution mitigation. According to Jaillon et al. [34], prefabrication application increased the average construction and demolition waste reduction level to 52%. On the one hand, the application of prefabricated construction combined with some emerging technologies, such as Building Information Modelling (BIM, a developing technology to form, organize, and manage throughout the construction project [35], radio frequency identification (RFID, a technology that used radio waves to identify objects [36], and Internet of Things (IoT, a new technology paradigm that was conceived to realize the interaction of machines and devices around the world [37]), reduced the production of construction waste at sources [5,38]. On the other hand, during the manufacturing stage, a large amount of wet work was transferred to the factory, prefabricated components were produced in a mechanized, standardized, and intelligent production line, resulting in a significant reduction in waste generation [39]. The air pollution produced by conventional construction methods involves carbon emission and on-site dust. Numerous scholars have compared the lifecycle greenhouse gas (GHG) emission of the prefabricated building with that of conventional building and revealed that prefabricated construction methods reduced GHG emissions [5,40,41]. Some studies even integrated digital technologies to achieve real-time monitoring of carbon and GHG emissions. For example, Liu et al. [42] developed a real-time carbon emission monitoring system for the entire industrial chain of prefabricated buildings that used five types of hardware to automatically collect data and could be simultaneously adapted to computer desktop platforms, browsers, and mobile phone applications. Besides, the generation of dust could be significantly mitigated by adopting prefabricated construction [43], avoiding affect the surrounding environment and public health [44,45]. Furthermore, Tsoka et al. [46] compared the energy performance of the conventional and prefabricated building and proved that the later one showed significant advantages. Currently, some researchers started paying attention to the green design that integrated the digital technology of modular buildings to achieve sustainability at the early design stage and contribute to the whole building cycle [47,48]. The early green design also benefited future modules’ reuse [49], which was one of the most important strengths of prefabricated construction. Few researchers have already begun exploring specific strategies for recycling and readoption in prefabricated projects [50,51].

However, despite the environmental benefits of prefabrication application, some researches also evidenced that transporting steel structures would produce more GHG emissions than prefabricated concrete and timber structures [52]. Also, the grating use of electricity in prefabricated construction would cause adverse impacts on eutrophication and water intake [53,54], which should be further considered in future prefabrication studies.

### 4.2. Economic Sustainability

The economic sustainability of prefabrication in construction was discussed in three perspectives: building quality, construction productivity, and lifecycle cost.

#### 4.2.1. Building Quality

##### Quality Control

Quality control was an essential factor that affected the safety of construction onsite. The factors (see Table 1), such as complex working conditions [55], weak safety awareness [56], and lack of quality control, may lead to accidents [57]. Compared to traditional construction methods, prefabrication could achieve better quality control [58]. The quality control of prefabricated components mentioned most in existing studies is reflected in the production stage because the automatic production lines replace manual operations and thus reduce manual errors. The introduction of the Design for Manufacture and Assembly (DfMA, a mature principle in the manufacturing industry that integrated the design for manufacture and design for assembly) in the design stage can also improve the quality of prefabrication [59,60]. From the transportation to the installation stage, various measures were conducted with the aim of protecting components, encompassing monitoring tools (e.g., Internet of Things) to check the status of components [19], and additional protection of the loading and fixation of each element in transporting [61]. Moreover, the collaboration of suppliers and contractors have also contributed significantly to achieve better quality control [19].

##### Quality Defects

There was a minority of scholars who still insisted on some barriers that occurred in different stages that might influence quality performance in some aspects (see Table 1). In the design stage, the factors influencing the quality of prefabrication were mainly reflected in two aspects, one being the lack of standards and specifications and the other being stakeholders’ participation in design works. Due to the decisive influence of design, the mistakes that occurred in the design stage would result in serious quality problems in subsequent processes, such as joint failure. These mistakes might result in design change, increased rework in prefabrication housing production (PHP), and higher costs [74]. During the manufacturing and transportation stage, the quality defects were mainly caused by technics (e.g., the unreasonable connection of joints) and uncertain surrounding environment (e.g., the dynamic loading of components during road transportation), further decreasing safety performance and increasing the cost of building components [70]. Taking the transportation stage as an example, Godbole et al. [73] explored the impact of dynamic loading during road transportation on prefabricated components. The results revealed that dynamic loading on the truck-trailer might trigger a weak connection of joints and even cause damage. In the installation stage, the quality influencing factors could be divided into three aspects: accelerating schedule, improving assembly rate, and inadequate stakeholders’ skills. Quality defects in this stage even increased the incidence of safety accidents [20].

To reduce quality problems, future studies should pay more attention to perfect design works, not only in requiring consistent standards and specifications, but also in improving the professional skills of designers and strengthening the collaboration between participants. Also, the integration of information technology (e.g., RFID) should be explored more to achieve real-time performance monitoring [75]. In addition, due to the differentiating influences produced by different stakeholders, it is also suggested to establish a responsibility recovery system to clarify the quality responsibility of each party and improve the quality management system to ensure the quality and safety of prefabricated buildings [76].

#### 4.2.2. Construction Productivity

##### Productivity Improvement

On the economic sustainability performance level, the framework indicated that productivity performance in prefabricated construction exists differences. A large proportion of researchers have claimed that prefabrication could effectively improve productivity, as shown in Table 2; the main reason was the support of information technology [62,77,78]. BIM technology has been frequently integrated adopted with other information technology, such as RFID [79], sensor technology [80], and Geographic Information Systems (GIS) [81], for it could simplify the procurement process of prefabricated components, improve information flow and the productivity of workflow between the designers and contractors [82,83,84]. Since prefabricated components were manufactured in the off-site environment, the work teams could solve the resource planning problems by cross-training to form multi-skilled resources, including workforce variation and shortage of skilled resources, which improved productivity and decreased fragmentation in prefabricated construction [85]. Besides, technological problems could be solved through production engineering innovation. For example, Sabet and Chong [84] proposed an integrated framework that conceptualized and clarified the possibility and functions of BIM and prefabricated construction interaction that could improve productivity based on scoping review. The higher quality prefabricated components could be obtained in a controlled factory environment, which were the prerequisites for productivity and efficiency improvement [64,86,87,88].

##### Schedule Delay

A few scholars have held opposite views that prefabricated construction could lead to schedule delays [94]. The key issues that contributed to schedule delay could be reflected in inflexible data/information exchange. The fragmentation, discontinuity, and poor interoperability of prefabricated construction was the major bottleneck that impeded the adoption of prefabrication in construction [67,93,95]. To address these problems, some researchers proposed that design information exchange should be considered not only during the design and manufacturing stages but throughout the whole construction process [96]. In addition, an integrated supply chain management with tremendous benefits to the environment, economy, and society [62,97] has been introduced. As an integrated cross-enterprise support approach, it supported the information sharing and collaboration between different parties and further propelled the establishment of risk-sharing and profit allocation mechanisms to achieve a better-integrated supply chain management [95,98]. At present, BIM has been popularly applied as a real-time information platform that provided real-time supervision to remove these obstacles [96,98,99,100]. However, in many developing countries (e.g., China), the reality was that the existing technologies had not synchronized the BIM platform into a project [96].

#### 4.2.3. Lifecycle Cost

##### High Capital Cost

Existing studies found that the capital cost is higher than conventional construction methods, which has become the most significant factor in affecting the willingness of stakeholders to adopt construction methods [69,87,101]. Table 3 depicts a summation of the key factors that cause high capital cost.

Deepening design cost. Prefabrication necessitated a more detailed design in some respects than conventional construction [59,112]. Even though the prefabrication design was standardized, the cost of the deepening design was high, increasing the capital cost [113]. Though some scholars proposed to decrease the deepening design cost [61,69], research on how to reduce the high costs was still rare.

Risk cost of components’ transportation and installation. Different from the transportation stage of conventional construction, the heavy and bulky prefabricated components resulted in more difficulties and higher expenses in prefabrication construction [66,69]. Besides transporting, components assembly was also an essential task in prefabricated construction [104,114], resulting in higher assembly onsite costs. Accordingly, significant efforts have been paid on how to optimize the transport route and layout on-site. For example, Ning and Lam [115] used a modified Pareto-based ant colony optimization algorithm and multi-objective optimization to optimize the construction site layout, which not only optimized the cost but also ensured safety on site.

Lack of market scale. The cost of precast components was also high due to the lack of scale economy [69,116]. Scale economy was challenging to achieve because of the lack of codes and standards for assembly-type production and prefabricated components suppliers in some jurisdictions (e.g., Hong Kong, mainland China). Scholars proposed the establishment of codes and standards according to the local conditions [110] and the enhancement of incentives [111].

High cost in other aspects. Apart from the cost increment mentioned above, the expenses in the other aspects were also responsible for the high capital cost, involving the costs of machines [104], materials [61], and laborers [66]. In addition, the special costs involved in prefabricated buildings should also be taken into consideration, such as design consulting fees and detailed design fees for joint performance [61,66,108].

##### Low Lifecycle Cost

Although a large number of scholars evidenced that the construction cost of prefabricated buildings was higher than that of conventional buildings [96], the result was the opposite when considering the cost from the perspective of the whole lifecycle of buildings. Despite the incremental cost in the construction stage, the advantages of standardized design, lower thermal energy consumption, convenient removal of components, fewer remnant materials [34], and other factors (see Table 3) occurring in other stages effectively reduced the lifecycle cost.

Profitability was one of the main concerns of the contractors. The high capital cost and unclear benefit justification had posed obstacles to the adoption and advancement of prefabricated construction [24,61], which should be further investigated in future research.

### 4.3. Social Sustainability

#### 4.3.1. Occupational Safety and Health

Occupational safety and health were considered significant aspects of risk and challenge in the construction workplace [117,118]. Various factors caused safety risks in the conventional construction site, such as massive labor inputs [119], complicated construction environment [120], and works’ potential safety hazards [55], which were believed to have been eliminated and improved in the prefabricated construction [121]. Shi et al. [24] compared the safety and health performance of prefabricated and conventional construction through field observation and interviews. They stated the hazards of manual handling in column and formwork installation and the exposure to chemicals in the curing process. Jeong et al. [122] evaluated accident cases that occurred in prefabricated construction projects in the United States and indicated that the familiar working environment, less high-altitude operations, and less exposure to bad weather were significantly beneficial to ensure occupational safety and health in modular projects. Their opinion echoed the arguments proposed by [123]. Murali and Sambath [123] also believed that the reduction of construction dust, noise, and other pollutants in prefabricated construction sites only protected the workers but also the surrounding communities. Moreover, it is noted that the labor-intensive construction activities mainly threatened workers’ safety [58,124]. Therefore, other than the fact that complex assembly works, typically done at the ground level/off-site, could decrease aerial works and further avoid accidents, fewer labor inputs in prefabricated construction could also reduce safety accidents onsite and contribute to sustainability development [125]. Emerging technologies, such as IoT and 3D visualization, have also been involved in the prefabricated construction process to achieve better safety control [126,127].

However, some long-existing hazard causes, like falling, were still the biggest threat to employee safety in prefabricated projects [20]. Achieving the development of falling protection system for working from height, stability of temporary loading platform, and safe usage of special equipment should be paid attention in future research.

#### 4.3.2. Social Climates and Attitudes

Social climate and attitudes played an important role in the development of prefabrication [128,129,130], especially the attitudes of governments and developers [72,128]. In the initial stage of prefabricated development, the government played a leading and facilitating role in introducing prefabrication into the construction market. One of the key reasons that the acceptance of prefabrication was still low [88] was that developers tended to pay more attention to clear economic benefits. The government, therefore, has formulated not only mandatory policies but also incentives [22,131] to encourage enterprises to adopt prefabrication. Moreover, the role of public opinions (e.g., customers’ opinions) in the adoption of prefabrication also could not be ignored [132]. However, few studies have been conducted on this aspect. It is suggested that future research should pay more attention to this area.

### 4.4. Technologies Development

Previous studies had indicated several advantages of the prefabricated construction technology, compared with the conventional technology, such as reducing reliance on the site labor force and improving the working condition and safety level [133], increasing the controllability of the entire project and achieving higher building quality [134], reducing construction waste and realizing life-cycle environmental sustainability [6], shortening the construction time and enhancing the working efficiency through operating simultaneously onsite and in the factory [135]. More technical studies were designed to examine and improve the structural performance of the prefabricated components and buildings for practice. For example, Hou et al. [136] conducted eight tests to explore the axial stability performance of the modular multi-column wall and made design recommendations based on the results. Taking high-rise hotel buildings as objects, Liu et al. [112] analyzed the mechanical properties, failure mechanism, and elastoplastic development principles of the structure through elastoplastic design examine and proposed an improved high-rise steel frame prefabricated structure with diagonal braces. Some researchers focused on fire safety and concrete materials adoption [16]. The modular connection performance, as a unique problem of prefabricated buildings compared to traditional buildings, had also received attention from researchers [137]. In addition, due to the characteristic of the high standard, many studies proved that the prefabricated construction technology was more suitable and valuable to combine smart and digital technologies, such as 3D scanning, BIM, and artificial intelligence [138,139,140,141]. Also, the more streamlined process of MiC made automated construction more likely to be realized [142]. The sustainable demand for modern buildings [143] and the wide promotion of innovative construction [144] had further brought promising environmental opportunities for the sustainable development of prefabricated construction.

However, since prefabricated construction technology is a developing technology, it has some existing technical issues. The transportation and logistic problem was one of the most concerning challenges. Extra high-quality protection was needed during transporting units to the construction site, and additional consideration for transportation regulations was required [145]. The logistics could be complex, and damages might occur during the delivery [146]. In addition, the inspection of modular production for the construction site could be complex because the modules were built in factories [147], which may influence the accuracy and completion of the modules [148]. Due to the low feasibility of the MiC project, intense coordination was significant to ensure that fabrication, transportation, and erection occur in sequence with minimal delays. Hence, high information exchange is needed [149]. However, efficient, complete, and timely means were still lacking in practice [147]. Moreover, the hoisting issues [150] and high-building worrisome performance [146] still required more examinations and suitable solutions. Though some researchers have paid attention to these issues, more studies are required.

### 4.5. Strategies for Promoting Prefabrication

#### 4.5.1. Mandatory Policy

Mandatory policy in this paper refers to the policy with legal character implemented under the compulsion of the government, which can be reflected in three aspects: materials and structures used, land transfer, and evaluation criterion [151]. Concerning materials and structures used, each country or region has put forward its corresponding mandatory requirements. For instance, prefabricated prefinished volumetric construction (PPVC) has been mandatorily adopted in non-landed residential government land sale sites in Japan and Singapore. In Hong Kong, a precast façade had been mandatorily used in all standard domestic blocks of public housings [152]. China used the “assembly rate” as the main evaluation basis for achieving the planning goals. Although the implementation effects of mandatory policies vary among countries, the most frequently used mandatory policy was related to “materials & structures used” and “evaluation criterion”. The mandatory policies played a significant role not only in prefabrication promotion, early-stage development, and direction guidance [153] but also in investment risk [71].

#### 4.5.2. Incentives

Incentives in this paper refer to the measures with an incentive character that governments formulated in order to encourage stakeholders to adopt prefabrication and can be categorized into economic incentives and non-economic incentives. Economic incentives, such as financial subsidy, tax allowance, land ratification policy, credit incentives, loan incentives, and gross floor area concessions, significantly improved the participants’ willingness to use prefabrication [14]. The aspects of non-economic incentives mainly involved benefits in transportation, reputation, and the approval process [131,154]. Although government incentives could promote the development of prefabricated construction to a certain extent in the initial stage, in the long run, it was the construction cost rather than government incentives that could determine whether companies employ prefabrication in the projects [155].

### 4.6. Future Research Directions

**Figure 3:**
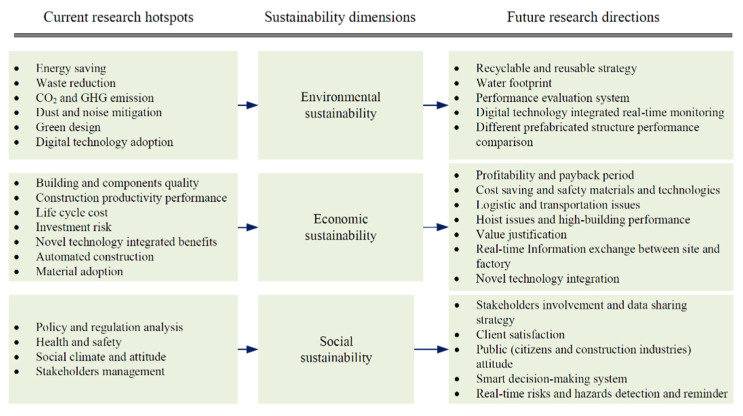
Current research domains and future directions of prefabrication towards sustainability.

Based on the in-depth analysis, a framework linking the current research status and future research directions was developed, as shown in Figure 3.

#### 4.6.1. Environmental Sustainability Research Directions

The environmental advantage is a fundamental reason for the promotion of prefabricated in the construction industry. As discussed, the existing research covers various aspects of environmental sustainability of the prefabricated construction, such as energy-saving, waste reduction, life cycle performance, and so on. However, research on water footprints is still scarce. Besides, although a few researchers have begun discussing and proposing strategies for prefabricated green design [48], recycling [156], and reuse [49], the reusable issue that seriously affects environmental and economic benefits still needs further exploration. Moreover, prefabricated buildings of different structural types may have different performances. For example, Zhou and Yang [52] argued that the transportation process could cause higher GHG emissions than conventional construction when adopting modular steel construction. Thus, comparative studies of the performance of prefabricated construction of different structural types also need attention in future research. In addition, digital technology has gradually been applied to the field of construction, including prefabricated construction. The application of BIM, sensors, Virtual Reality, Augmented Reality, and other emerging technologies make it possible to achieve real-time monitoring and managing the entire life cycle of prefabricated construction. Future research may explore specific strategies to combine technological innovation and development with prefabrication to further improve environmental sustainability.

#### 4.6.2. Economic Sustainability Research Directions

Existing studies have revealed many factors that contributed to high capital costs, but suggestions on how to save existing costs have yet to be explored. Thus, future studies could investigate and propose strategies to save costs from different perspectives. For example, the materials and technologies used in construction are the most expensive, considering a cost-saving perspective. In addition to the high cost of materials and technology, the unformed market scale also results in high capital costs. There have been numerous researches on the relationship of government with developers and contractors in the prefabrication market. However, as the demand side’s main body, customers are seldom considered in the prefabricated market research. Thus, it is suggested to study the prefabrication market, which should involve all stakeholders, not only the main body of the supply side. The safety performance also requires more attention in the material and technology exploration studies. Moreover, hoist issues of the large components and the stability of the high-level buildings still require more examination and practical studies for improvement. Besides, it has been proved that the logistic issues in prefabricated projects, especially multiregional projects, are complex and significantly influence the project schedule and cost [157]. Also, the barriers to information communication between the construction site and the factory affect the quality assurance and project progress. Future studies should consider integrating the novel technologies in construction management and propose optimization solutions. Besides, though digital technologies offer new opportunities in various respects to the construction project, in some developing countries, the benefits of digital technologies in construction are still rhetoric, with numerous barriers in its practical application [158,159]. One of the most critical barriers is the negative attitudes of stakeholders towards data sharing, which further affects technology advancement [159,160]. Therefore, it is of great importance to explore the strategies of inspiring stakeholders to involve and share the data in future studies. Furthermore, the uncertain profitability and payback period have posed obstacles to expanding the prefabricated construction market. Prospective studies could consider assessing the profitability and payback period and justifying the value of adopting prefabricated construction.

#### 4.6.3. Social Sustainability Research Directions

Although the governments in developing countries have promulgated a series of mandatory and incentive policies, the development of the prefabrication is far behind that of developed countries. To formulate reasonable policies, the effectiveness of the prefabrication policy should be quantified. Existing researches on methods to study prefabrication policy mainly encompass content analysis [161], evolutionary game [110,128], and social network analysis [76,162], which all fail to quantify the effectiveness of prefabrication policy appropriately. The bottom-up analysis based on stakeholders with the assistance of computer tools should be considered within the scope of future research, such as agent-based modeling (ABM) [163]. In addition, the stakeholders evolved in existing research mainly include the government, developer, supplier, and contractor, considering the customer as the main body of the demand side, whose attitude is also critical to the implementation of prefabrication. Thus, the public attitudes and involvement and client satisfaction should be concerning in future studies. Moreover, concerning factors and risks influencing prefabrication implementation, the current research status is mainly stuck in the stage of factors identification. The interrelationships between various factors still require attention. Besides, the protection system for working at height, the stability of temporary loading platforms, and safe usage of special equipment are urgently awaiting exploration and development. Novel technologies could be considered to apply in building the real-time risks and hazards detection and reminder system. The technologies could also be employed to support smart decision-making in future efforts.

Furthermore, in terms of performance evaluation of prefabrication, the current research areas include environmental performance, economic performance, and social performance, all of which were separately evaluated and neglected their interactions. Thus, a holistic performance evaluation system could also be constructed in future research.

## 5. Conclusions

The construction industry has been long recognized as posing heavy pressures on the environment. Due to the increasing demand for environmental protection, sustainable development, and modern buildings, prefabricated technology has gradually been noticed and promoted in the construction industry.

Although multitudinous studies have explored different aspects of the prefabricated construction, a systematic review that synthesized environmental, economic, and social sustainability dimensions of the prefabricated construction remains overdue. This study aims to thoroughly explore the status quo of prefabrication implementation in construction industries, analyze the different sustainable development dimensions, and provide potential directions for future research to fill this research gap.

Through the comprehensive review of 768 papers with the assistance of Nvivo, a research foci framework that represented the body of knowledge in prefabrication in construction was constructed. Five levels identified in the framework were advantages, hindrances, stakeholders, promotion policies, and strategy spectrum. The identified parameters were further incorporated into environmental, economic, and social sustainability dimensions, as well as the technologies development and promotion strategies with in-depth analyses. Based on the discussions, the framework linking current status and future research directions towards sustainability were delivered in this study. The main findings and future research recommendations are presented as follows. 

In the environmental sustainability dimension, the application of prefabrication, along with information technology and environmental-friendly materials, has produced a significant positive impact, which can be reflected in energy saving, waste reduction, CO_2_ and GHG emission reduction, dust and noise mitigation, and green design. In the economic sustainability dimension, the introduction of DfMA can effectively improve the quality of construction, the application of integrated information technologies (e.g., BIM and RFID) contributes to the real-time status information sharing of components among stakeholders and improve the productivity and the lifecycle cost saving in other phases offset the incremental construction cost. Whereas, some barriers that caused quality defects, schedule delays, high capital costs, and uncertain investment risks should not be neglected. In the social sustainability dimension, prefabrication implementation decreases the complex and aerial works, improves the safety performance onsite, and low labor input solves the problem of labor shortage, producing significant positive impacts on social sustainability.

The potential future research directions of the prefabrication studies are the recyclable and reusable strategies, water footprint, performance evaluation system, digital technology integrated real-time monitoring, and different prefabricated structure performance comparison in the environmental sustainability dimension. The areas that require further exploration in the economic sustainability dimension are profitability and payback period, cost-saving and safety materials and technologies, logistic and transportation issues, hoist issues and high-building performance, value justification, real-time information exchange between site and factory, and novel technology integration. In terms of social sustainability, the policy effectiveness quantification, client satisfaction, public attitude and involvement, smart decision-making system, and real-time risks and hazards detection and reminder system are the areas to be investigated.

The findings of this study help readers holistically understand the current status of prefabrication implementation, including its technology development, impacts on the sustainable development of the construction industry, promotion strategies, and future research directions. The study makes contributions to both the body of knowledge and various stakeholders.

The limitation of this study is that, since the study only analyzed the articles published in English collected from WoS, some relevant content may possibly not be involved in this study. Hence, the discussions of this paper should be interpreted regarding this limitation.

## Figures and Tables

**Table 1:** Existing study on the key factors influencing the quality of prefabrication.

Categories	Influence Factors	References
Improve quality	Design for Manufacture and Assembly (DFMA)	[59,60]
	Win-win relationship between supplier and contractor	[62]
	Monitoring tools for monitoring and checking the status and quality problems	[19,63]
	Additional protection of loading and fixation	[61]
	Concentrating on each single element	[61]
	Quality supervision	[64]
Quality issues	Accelerate the process	[65,66]
	Excessive pursuit of assembly rate	[66]
	Increased use of prefabrication for a relatively shorter time period	[22]
	Incompetent design	[67]
	Technical issues	[68,69,70,71]
	Lack of competence in the assembly	[66]
	Lack of standards and specifications	[68,69]
	Stakeholders’ experience and skills	[67]
	The knowledge of workers, designers, manufacturers, and assemblers	[66,72]
	Dynamic loading of components during road transportation	[73]

**Table 2:** Existing study on the key factors influencing the productivity of prefabrication.

	Influence Factors	References
Improve productivity	Information Technology	[62,77,78,84]
	Production engineering innovation	[87]
	Multi-skilling	[85]
	Design standardization, modularization and recycling	[87]
	Better quality achieved at the factory production	[88]
	Increase resources	[87]
Schedule delay	Slow quality inspection procedures	[89,90]
	Misplacement on the storage site resulting from carelessness	[89,90]
	Owner crane breakdown and maintenance	[67]
	Inefficient design data transition	[67]
	Project scale, resources, and management	[67]
	Inefficient verification of precast components because of ambiguous labels	[67]
	Lack of competence in the assembly	[66]
	Long design time	[2,91]
	Inflexible for design change	[92,93]
	Inefficiency of design approval	[67,89]
	Delay of the delivery of precast element to site	[90,92]
	Low information interoperability between different enterprise resource planning systems	[67]
	Design information gap between designer and manufacturer	[67]
	Installation error of precast elements	[67]
	Logistics information inconsistency because of human errors	[67]

**Table 3:** Existing study on the key factors influencing the cost of prefabrication.

Categories	Influence Factors	References
Increased cost	Design diversity & complexity	[2,102,103,104]
	Lack of codes and standards	[31,68]
	Unknown techniques	[31,87,104]
	Well-proven methods and materials	[87]
	Aesthetics	[87]
	Maintenance complexity	[87]
	Quality impression	[31,87,105]
	Supply chain issues	[31,87]
	Additional transportation cost	[69]
	Additional procurement cost	[61,66]
	Highly skilled workers	[31,61,66,106]
	Complex techniques	[24,61]
	Extra labor cost on checking, counting, and sorting raw materials	[61]
	Long lead-in times	[2,24]
	Design change	[2,102]
	Occupying extra space for the accommodation of precast components	[61]
	Additional use of tower cranes (vertical transportation)	[61]
	High employee training cost	[61]
	Lack of knowledge and understanding	[31,96]
Decreased cost	Decreased labor	[14]
	Cheaper labor rates	[2,107]
	High thermal efficiency	[14]
	Fewer site materials	[34]
	Increased productivity	[108]
	Avoidance of construction site hindrances	[61]
	Decreased management cost	[61]
	Faster project delivery	[61]
	Decreased transportation cost for materials & waste	[34,109]
	Decreased waste disposal cost	[34]
	Reduction of formwork	[61]
	Controlled quality	[61]
	Lower maintenance and repair expenses	[61]
	Incentive mechanisms	[110,111]
